# Artificial Sweetened Beverages and Pediatric Obesity: The Controversy Continues

**DOI:** 10.3390/children1010031

**Published:** 2014-05-28

**Authors:** Peter N Freswick

**Affiliations:** Department of Pediatric Gastroenterology, Vanderbilt University, Nashville, TN 37232, USA; E-Mail: peter.n.freswick@vanderbilt.edu; Tel.: +1-(615) 322-7449

**Keywords:** artificial sweetener, weight gain, obesity, pediatrics

## Abstract

The pediatric obesity epidemic has gathered public and political interest recently. People often choose “diet” or artificial sweetened beverages (ASB) to combat this epidemic, but the obesity incidence continues to rise. First, I review the pediatric studies on the effect of ASB consumption with subsequent food intake. Next, I present pediatric studies of chronic ASB consumption and weight change. Some epidemiologic pediatric studies have supported an association between artificial sweetener use and increased BMI but cannot prove causation. Randomized control trials have provided some evidence of weight loss with ASB ingestion among children, but study limitations may minimize these conclusions. Finally, I summarize the possible mechanisms that may drive potential effects of artificial sweeteners.

## 1. Introduction

Obesity among the young in the United States has tripled in the last 30 years [1] reaching epidemic proportions, with 17% of children and adolescents now classified as obese [2]. Childhood obesity contributes to dyslipidemia [3,4,5], hypertension [6,7], non-insulin-dependent diabetes mellitus [1,6,8], and nonalcoholic fatty liver disease [9]. Though many factors contribute to pediatric obesity, excess sugar ingestion may play a prominent role in excessive weight gain among youth [10,11]. Children, especially adolescent males, consume excessive added sugar from nutritive sweetened beverages (NSBs) such as regular soda and juice [12,13].

Substituting NSBs with ASBs such as diet soda presents a logical strategy for preventing and treating obesity. As the diet industry champions artificial sweeteners as “health food” without calories, we view ASBs as the ideal NSB substitute [14]. Despite FDA approval, the benefits of artificial sweeteners on our health have been researched and questioned for decades [15]. Adult epidemiologic studies first associated ASB consumption with weight gain [16,17,18], though another study negated these findings [19]. In light of recently published randomized control trials on ASB in children [20,21], this review summarizes ASB use and its impact on pediatric obesity, food intake, and brain reward activation.

## 2. Artificial Sweetened Beverages and Subsequent Food Intake

Several studies have investigated the association of artificial sweetener ingestion on subsequent caloric intake. A Compensation Index (COMPX) score reflects the difference of caloric intake during *ad-lib* meals across two preload conditions [22] using the following formula:

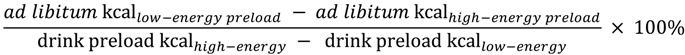
(1)


This score characterizes subjects from negative compensation (*i.e*., consuming more at *ad-lib* meal after drinking high-calorie preload) to positive compensation (*i.e*., consuming less at *ad-lib* meal after drinking high-calorie preload). A COMPX was computed if not originally reported in the following reviewed studies.

Johnson *et al*. (2006) [23] found partial compensation (mean 48.6% compensation) during an *ad-lib* meal 30 min after ASB *versus* NSB ingestion in 342 children (5–11 years old). Interestingly, younger age predicted significantly greater caloric compensation (R^2^ = 0.2, *p* < 0.05) with 5-year-old girls demonstrating 80% compensation. Faith *et al*. (2004) [24] recruited 32 sibling pairs (3–7 years old) to investigate familial compensation correlation after ASB and NSB ingestion. They concluded that though relatedness did not predict COMPX scores, the children demonstrated significantly more *ad-lib* meal caloric intake (average 152 kcal) after low-energy preload (103.6% compensation, *p* < 0.001). Bellissimo *et al*. [25] investigated caloric intake during an *ad-lib* pizza meal 30 min after sucralose *versus* glucose preload ingested by 9- to 14-year-olds undergoing fitness testing and moderate intensity exercise. The boys demonstrated 94% compensation (*p* < 0.05) after ASB *versus* NSB ingestion, though physical activity and fitness did not correlate with caloric compensation. Bellissimo *et al*. [26] also investigated caloric intake during an *ad-lib* pizza meal with or without TV viewing 30 min after sucralose *versus* glucose preload ingested by 9- to 14-year-old boys (2 × 2 crossover design). The boys demonstrated 112% caloric compensation with no TV viewing, but only partial compensation (66%) if watching TV. TV viewing increased caloric intake irrespective preload type (*p* < 0.01).

When subjects eat a test meal more than 30 min after preload beverage, compensation is not observed. Birch *et al*. (1989) [27] found that children (2–5 years old) demonstrated partial caloric compensation during an *ad-lib* snack immediately and 30 min after drinking an ASB *versus* NSB (69% and 61% compensation respectively, *p* < 0.05). When offering an *ad-lib* snack 60 min after sweetened preload, no compensation was observed (34% compensation, *p* > 0.05). Likewise, among 20 children 9–10 years old, Anderson *et al*. (1989) [28] found no difference in energy intake during an *ad-lib* meal 90 min after a sucrose or aspartame sweetened beverage preload (5% compensation, *p* > 0.05).

### Summary

These studies demonstrate partial to full caloric compensation during an *ad-lib* meal served 0 to 30 min after ASB *versus* NSB preload. Younger children may demonstrate more complete compensation, though more recent studies by Bellissimo *et al*. [25,26] conclude that older children also demonstrate complete compensation. Caloric compensation was not observed during a test meal served more than 30 min after beverage ingestion. These studies do not, of course, describe the effect of chronic consumption of ASBs on food intake or obesity.

## 3. Observational Studies of Artificial Sweetened Beverages

### 3.1. Prospective Cohort Studies

The majority of pediatric prospective cohort studies have found a positive or neutral correlation between weight gain and ASB intake. Vanselow *et al*. (2009) [29] followed 2294 adolescents (mean age 14.9 years old) of diverse background over five years and found that ASB consumption positively correlated with weight gain, though the significance was lost after adjusting for the child’s dieting behavior. Blum *et al*. (2005) [30] observed that ASB consumption correlated with increased BMI among 164 children (8–10 years old) over an observational period of 2 years, while Berkey *et al.*’s larger cohort of 11,654 children (9–14 years old) found this positive correlation was significant only among boys, not girls [31]. A 10-year prospective study that periodically surveyed 2,371 girls (9–10 years old) of diverse background observed significantly increased caloric intake with increasing ASB and NSB consumption, though only NSB consumption correlated with increased BMI [32].

Prospective studies of younger children have found similar results. Johnson *et al*. (2007) [33] observed 1,203 children (5–7 years old) for 4 years and concluded that ASB ingestion positively correlated with fat mass change on dual x-ray absorptiometry. A smaller study that followed 177 children (3 years old) for three years demonstrated that waist circumference was higher with increased NSB intake but not ASB intake; however, neither ASB nor NSB intake predicted BMI change [34].

One observational study demonstrated an inverse relationship between ASB ingestion and BMI. After following 548 children (mean age 11.7 years) over 19 months, Ludwig *et al*. (2001) [35] noted that each additional NSB serving per day was associated with frequency of obesity (OR = 1.6, 95% CI 1.1-2.24, p = 0.02), whereas ASB consumption was negatively associated with obesity incidence. Baseline ASB ingestion was not associated with obesity incidence.

### 3.2. Cross-Sectional Studies

Three pediatric cross-sectional studies have demonstrated similar findings. O’Connor *et al*. (2006) [36] analyzed National Health and Nutrition Examination Survey data on 1572 children (2–5 years old) and observed no association between type of beverage consumption (including ASB and NSB) and BMI. Forshee *et al*. (2003) [37] surveyed 3,311 children (6–19 years old) and found that BMI correlated with ASB but not NSB ingestion. Another survey of 319 children ages 11–13 years concluded that both ASB and NSB ingestion were associated with increased BMI [38].

### 3.3. Summary

Of the 10 observational studies reviewed here, one study demonstrated that ASBs are associated with weight loss in children while most other studies demonstrate neutral association. Positive association of ASB with weight gain where found in one epidemiologic study [37], one prospective study, and boys in a final prospective study [31]. Observational studies cannot investigate causality. Reverse causality, *i.e.*, individuals at higher risk for weight gain may choose to consume ASBs when attempting to lose weight, may pervasively bias observational studies [39]. Thus, considering the inconsistency of these findings and potential biases, causality of ASB ingestion and weight gain or loss is far from established.

## 4. Interventional Studies of Artificial Sweetened Beverages

To date, four pediatric randomized control trials have investigated the effects of artificial sweetener beverage ingestion on weight change. In the first pediatric randomized controlled trial investigating ASB and weight change, Williams *et al.* (2007) [40] restricted 38 obese girls (11–15 years old) to 1500 kcal per day (with three meals and two snacks) and randomized them to one free snack (*i.e*., one “unhealthy” snack including a caloric soft drink) or restricted snack (both snacks where healthy, and could include ASBs) per day. Though greater than 50% of subjects drank at least 3 NSB (free snack group) or ASB servings (restricted snack group) per week, investigators observed no BMI change between groups.

Ebbeling *et al*. (2006) [41] randomized 103 adolescents (age 13–18 years old, varying BMI) stratified by BMI and gender to an intervention group (ASBs delivered to home) or a control group (instructed to continue baseline beverage consumption habits) for 25 weeks. Between the two groups there was no significant net difference in weight despite 250 fewer calories ingested per day in the intervention group. However, a regression analysis demonstrated that higher baseline BMI correlated with greater weight loss in the ASB *versus* control group (*p* = 0.016, BMI decreased 0.08 kg/m^2^ with every 1 kg/m^2^ increase in baseline BMI). Subgroup analysis revealed that the greatest difference was among the six subjects with a BMI > 30 kg/m^2^. The ASB intervention group was contacted monthly to assess beverage satisfaction, beverage consumption, and give motivational counseling whereas the control group did not have these additional interventions thus potentially confounding results.

Ebbeling *et al*. (2012) [20] expanded their previous trial, randomizing 224 overweight and obese adolescents to an intervention group (bottled water and “diet beverages” delivered every 2 weeks) or a control group (continued baseline beverage consumption) for one year; then followed all participants for an additional one year. The investigators phoned the intervention group monthly (but not control group) and held three check-in visits to assess beverage consumption and provide motivation counseling. Investigators encouraged the intervention group to consume water instead of ASB; subsequently both water and ASB intake significantly increased by about 1 serving per day. There was no difference in BMI change at 1 year (−0.29 kg/m^2^ in interventional group, *p* = 0.36) or 2 years (+0.18 kg/m^2^, *p* = 0.68) in non-Hispanic adolescents. However, Hispanics in the intervention group demonstrated significantly decreased BMI at 1 year (−1.79 kg/m^2^, *p* = 0.007) and 2 years (−2.35 kg/m^2^, *p* = 0.01) in a subgroup analysis. Interestingly, the authors conducted a *post-hoc* analysis on Ludwig *et al.*’s (2001) [35] 19 month prospective observational study described previously, discovering significant effect modification to Hispanic adolescents with a positive association between NSB consumption and BMI (β = 0.63, *p* = 0.007) but observed no significant correlation between BMI and NSB consumption among the non-Hispanic adolescents.

De Ruyter *et al*. (2012) conducted a rigorously designed randomized, double blind, controlled trial allocating 641 healthy weight children (4–11 years old) to drink either an ASB (0 kcal, sweetened with sucralose and acesulfame) or NSB (104 kcal, sweetened with sucrose) during midmorning break for 18 months. After 18 months, a per-protocol analysis of the 74% subjects who completed the study demonstrated decreased BMI z-score change among the ASB *versus* NSB cohorts (-0.13 kg/m^2^, 95% CI -0.2 to -0.06, *p* = 0.001). However, investigators observed no difference in the intention-to-treat analysis (dropout of 136 subjects, mean difference 0.07 kg/m^2^, 95% CI −0.134 to 0.002, *p* = 0.06).

### Summary

These randomized interventional studies found a neutral or negative association between ASB ingestion and weight change. However, several limitations may affect their generalizability. Williams *et al*. (2007) [40] restricted both cohorts to 1500 kcal diets thus only investigated caloric efficiency (not potential compensation effects) of ASB *versus* NSB ingestion. Ebbeling *et al*. (2012) [20] encouraged water rather than ASB consumption and the intervention group increased consumption of both, thus only water may explain the observed weight loss. Finally, De Rutyer *et al*. (2012) [21] had a 26% dropout rate and gave children their ASB or NSB beverage during an caloric limited snack [42], not during a calorically unrestricted meal. Thus, though these studies generally favor ASB ingestion for preventing weight gain, their limitations may preclude definitive conclusions.

## 5. Mechanism of Artificial Sweeteners

To explain the possible paradoxical association of artificial sweeteners and weight gain, multiple mechanisms have been proposed. Artificial sweeteners may increase carbohydrate absorption [43,44]. Furthermore, adults may knowingly overcompensate caloric intake when consuming “healthy” artificial sweetened beverages. However, studies have not supported these hypotheses as major contributors to weight gain [45,46].

Artificial sweeteners may influence behavioral food intake. Food intake and overeating is influenced by a convergence of hedonic (pleasurable) and homeostatic processes in the brain [4]. Hedonic brain regions can promote eating despite the brain’s homeostatic inhibition modulated by feedback hormones such as insulin, leptin, peptide YY, and GLP-1 [47]. Increasing evidence suggests that artificial sweeteners may activate hedonic brain responses differently than nutritive sweeteners. Functional magnetic resonance imaging studies demonstrate that nutritive sweeteners elicit stronger brain stimulation in hedonic regions such as the caudate, putamen, nucleus accumbens, anterior cingulate cortex, and ventral tegmental area when compared to artificial sweeteners [48,49,50,51]. Therefore ASBs may decrease hedonic brain response when sweetness is disassociated from its historical energy content, thereby offering only partial but not complete activation of these brain regions. A subsequent feeling of unsatisfaction may fuel further food compensation [52].

## 6. Conclusion

Epidemiologic studies, both prospective and cross-sectional, generally support a positive or neutral association between ASB ingestion and BMI. One epidemiologic study (prospective cohort) demonstrated a negative association of ASB ingestion and BMI z-score change [35]. Epidemiologic studies, however, may fall victim to reverse causality [39]. Furthermore, to demonstrate a causal relationship between disease and environment, strength of association, consistency of findings, specificity of the association, temporality, dose-dependent biological gradient, plausibility, and coherence must all be demonstrated [53]. Though current epidemiological studies cannot support causality between ASB consumption and weight gain, most demonstrate an unfavorable association.

Interventional studies support a neutral or negative association between increased ASB ingestion and weight gain. These findings may be affected by limitations of these studies. Limitations include possible confounding effect of water on weight change [20], a calorically restricted diet [40], and test beverage administration between meals [21]. As many studies endorse partial or full caloric compensation when subjects ingest an ASB within 30 min of an *ad-lib* meal [23,24,25,26], future trials should especially avoid these later two limitations.

Pediatricians increasingly encounter obese patients with associated comorbidities. Scientific evidence does not currently provide sufficient evidence to endorse or restrict ASB consumption among children. Perhaps, unsweetening our youth’s diet may be a key to reversing the obesity epidemic [52]. Future studies are needed to investigate the effects of artificial sweeteners on human metabolic pathways, brain stimulation, and weight change.
